# Is revisional surgery mandatory when an unexpected sarcoma diagnosis is made following primary surgery?

**DOI:** 10.1186/s12957-015-0719-y

**Published:** 2015-10-24

**Authors:** Georgios Koulaxouzidis, Eugenia Schwarzkopf, Holger Bannasch, G. Björn Stark

**Affiliations:** Department of Plastic and Hand Surgery, University of Freiburg Medical Center, Freiburg, Germany; Comprehensive Cancer Center Freiburg (CCCF), Section Plastic and Reconstructive Tumour Surgery, University of Freiburg Medical Center, Freiburg, Germany

**Keywords:** Soft tissue sarcoma, Unplanned excision, Re-excision, Revisional surgery, Whoops procedure, Comprehensive cancer center

## Abstract

**Background:**

Soft tissue sarcomas (STS) are often diagnosed unexpectedly after surgery, and many excisions are incomplete. As histopathological assessments are challenging, patients later referred to comprehensive cancer centers (CCC) often come with an unclear status. This can make treatment planning problematic. We investigated the reliability of primary histopathological assessments, whether revisional surgery improved resection status, and the prognostic value of residual tumor at re-excision.

**Methods:**

We analyzed the demographic and clinical characteristics of all patients referred to our CCC between 2003 and 2013. We compared patients treated exclusively at our CCC with those who had primary surgery elsewhere, and focused on resection margins, re-excision type, residual tumor, resection status after re-excision, and oncological outcome.

**Results:**

Over half (*n* = 110) of all patients (*n* = 204) were referred from elsewhere. Seventy-one had undergone an excision without suspicion of malignancy. Resection status in referred patients was significantly inferior to the CCC group (*p* < 0.0001), although the latter had significantly more serious tumors and advanced disease stages (*p* < 0.05). The residual tumor rate was 53.13 %, with a significantly higher probability in an upper extremity (*p* = 0.001). Initial histopathological classification was misleading in 46.9 % of cases. Re-excision improved resection status in 69 % of cases. Residual tumor presumably leads to higher rates of local recurrence (*p* = 0.057) and significantly shorter times to recurrence (*p* < 0.05).

**Conclusions:**

Re-excision should always follow unplanned STS excisions. Resection margins and histopathological assessments from referring institutions are often unreliable and unsuitable for treatment planning. Residual tumor is a risk factor for earlier and more likely local recurrence.

## Background

It is generally recommended that soft tissue sarcomas (STS) should be treated in tumor centers [1–8]. Ideally, a patient should be referred to a tumor center when a sarcoma is suspected and before undergoing a biopsy or excision [9]. It is essential that a meticulous diagnostic and staging workup is performed and that a multidisciplinary tumor board draws up a treatment plan and considers a range of reconstructive surgeries [9–11].

However, soft tissue sarcomas are a heterogeneous group of rare malignant mesenchymal tumors with an annual incidence of two to three per 100,000 cases [12, 13]. It is estimated that every 200 to 300 lumps is a sarcoma. Given the rarity and diversity of these tumors, it is not surprising that excisions are often done without the preoperative suspicion of a malignant tumor, without appropriate preoperative imaging, without a sufficient biopsy or staging, and without regard for adequate resection margins. Such procedures are known as unplanned excisions [4, 14] or “whoops” procedures [2]. Radical tumor extirpation is the cornerstone of treatment [15, 16]. No other adjuvant or neo-adjuvant treatment provides adequate local tumor control [17]. Unplanned excisions are often incomplete, with residual tumor reported in 35 to 74 % of patients [3, 4, 14, 18–21]. These cases are associated with more mutilating surgery and higher treatment costs [6, 22]. Data on how re-excision affects oncological outcomes are conflicting. Principally, adequate re-excision has been said to improve local and systemic tumor control, or even overall survival, compared to single adequate primary tumor excision [23, 24]. Other studies, however, have shown higher local recurrence rates, impaired prognosis and increased rates of metastasis even after sufficient re-excision [3, 6, 25, 26].

Although re-excision is generally recommended [3, 4, 16, 18], clinical or radiological suspicion of residual tumor is rare and patients receive advice based on the treatment given and on the existing histopathological information. Reliable histopathological assessments of excised tissue or biopsy specimens suspected of sarcoma require a pathologist who has experience in that particular field. Therefore, even in comprehensive cancer centers (CCCs) like ours, which specializes in sarcoma treatment, external experts are regularly tasked with reference reviewing histopathological assessments. Previously published data show that the reliability of histopathological assessments performed by inexperienced pathologists is often questionable [27–29]. Initial tumor characteristics, such as size, location, and grading, as well as preoperative and postoperative radiation were found to be of no help in predicting the probability of a patient having residual tumor after an unplanned excision [4]. Additionally, cross-sectional imaging such as MRI does not predict residual tumor with sufficient sensitivity and specificity [30].

In our study, we evaluated the reliability of primary histopathological assessments produced after unplanned excisions that were performed in a non-CCC setting. Additionally, we assessed the improvement of the resection status by revisional surgery. Finally, we highlighted the prognostic value of residual tumor at re-excision for local and systemic tumor control.

## Methods

A retrospective chart review was performed for all patients who were referred to our center for definitive treatment of STS. Particular attention was paid to patients who had undergone a primary excision in a non-CCC setting. Patients treated between 1 January 2003 and 31 December 2013 were included. The follow-up period was extended until 30 April 2014. Only patients with complete data sets were included.

### Presentation status at referral and resection status

Patients who had previously undergone surgery were categorized according to their status at presentation:

-  Patients referred after an unplanned excision (“whoops” procedure)
-  Patients referred with local recurrence after a single excision or repeated excision
-  Patients referred after an incisional biopsy

Staging was performed according to the previous staging procedures and in line with the guidelines of our CCC. All patients underwent re-excision after their cases were presented to the multidisciplinary tumor board of our CCC. An individual therapy plan was drawn up at a second presentation to the board, with definitive histopathological assessment after re-excision.

The following data were collected: gender, age at diagnosis, location (extremity, trunk), size (<5 cm, ≥5 cm), depth (superficial or deep to the fascia), histotype, grade (FNCLCC grading), presentation status at referral, and primary resection margins (R0, R1, or R2 resection). Resection status at referral and after re-excision was compared to the resection status achieved in patients treated exclusively in our center.

### Residual tumor

After re-excision, a histopathological assessment was performed. If residual disease was found, we determined the improvement of resection status by re-excision. Characteristics of cases with and without residual tumor were assessed after patient categorization according to the resection status and tumor detection. Each patient was coded in terms of his or her primary resection status (R0, R1, or R2, external pathology), the resection status after re-excision (R0, R1, R2, CCC pathology with external expert review in individual cases), and the presence or absence of residual tumor at re-excision (0: no residual tumor found; 1: residual tumor found). For example, R1-R0-1 means that the patient was referred for definitive treatment after microscopic incomplete resection (R1 resection), that a complete resection (R0) was achieved after re-excision, and that residual tumor was found in the histopathological workup of the re-excision specimen. The surgical techniques used for the re-excision were also analyzed. For the referred patients, we determined the progression-free survival with regard to local recurrence and metastasis, and the disease-specific survival, based on the presence or absence of residual tumor.

### Local and systemic tumor control

Significance of local tumor recurrence based on residual tumor at re-excision was estimated with a Chi^2^ test. All groups were compared using a univariate test for relevant group characteristics and prognostic factors (gender, age, tumor grade, tumor size, tumor location, diagnosis, therapy). Dates of neoplastic events (local recurrence, distant metastasis) and survival status (alive with disease, AWD; no evidence of disease, NED; death from other causes, DOC; and died of disease, DOD) and dates of death and last follow-up were assessed.

### Statistics

For the statistical analysis of the categorical data, we used either a Chi^2^ test or Fisher’s exact test, depending on the number of cases compared. All groups and prognostic factors (gender, age, tumor grade, tumor size, tumor location, diagnosis, therapy) were analyzed by univariate analysis. Survival analysis data were presented as Kaplan–Meier curves. Comparison was done by log-rank test. A two-sided *p* value of <0.05 was assumed as significant.

## Results

From 1 January 2003 to 31 December 2013, 204 patients underwent surgery for STS at our plastic surgery institute. Of those, 110 patients (53.9 %) were referred to us after receiving therapy elsewhere.

### Presentation status at referral

Presentation status was established with certainty in 96 cases (87.3 %). A “whoops” procedure was performed in 71 cases (74 %). Recurrent local disease, either after a single excision or repeated excision, was found in 22 cases (22.9 %). Just three cases (3.1 %) were presented after incisional biopsy.

### Resection status

**Fig. 1:**
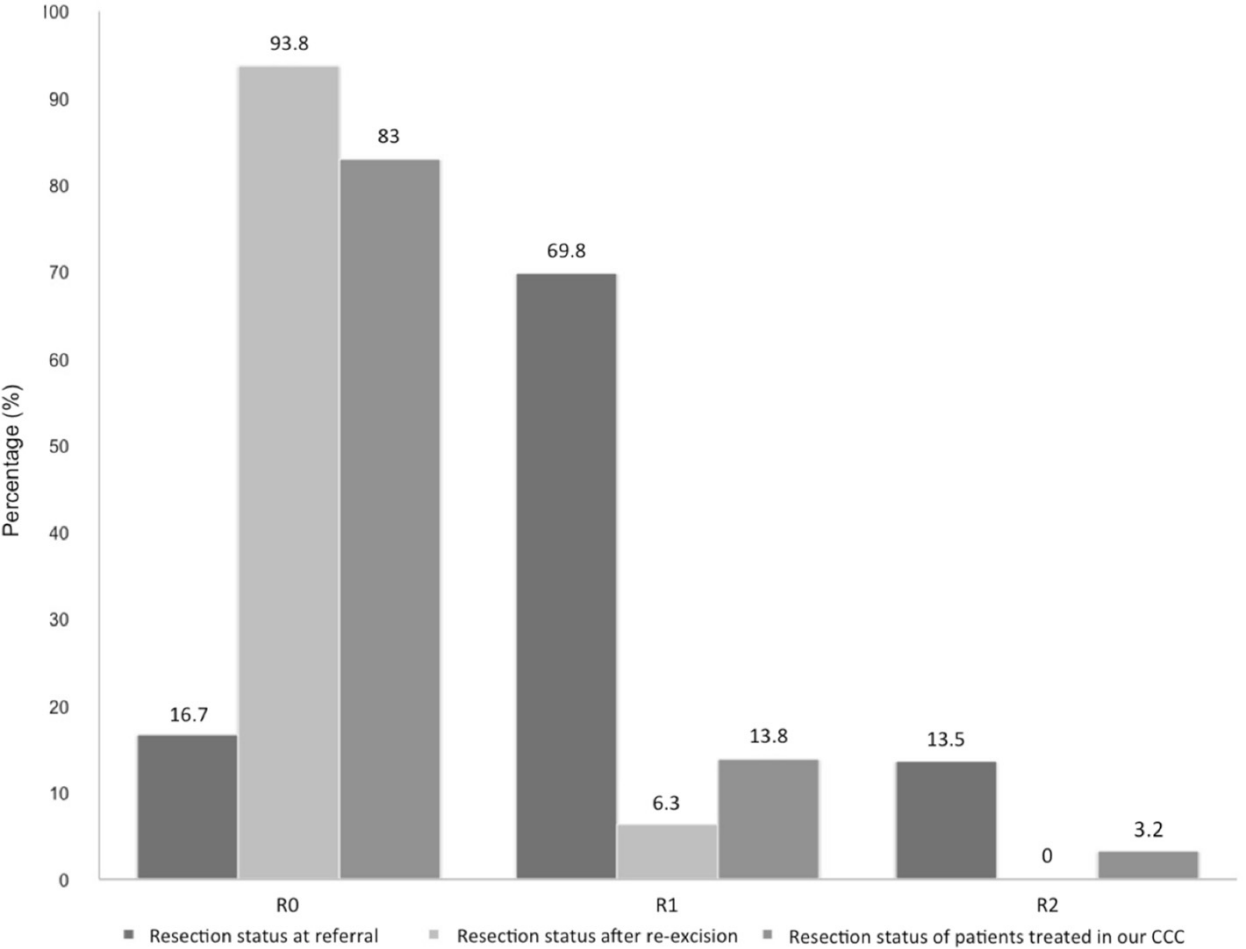
Resection status. Resection status was defined as complete resection (R0 resection), microscopic residual tumor (R1 resection), and macroscopic incomplete resection (R2 resection). It is displayed on the *x*-axis. Of the patients referred for definitive treatment with complete data sets (*n* = 96), 16.7 % had R0 resection status, 69.8 % had R1 resection status, and 13.5 % had R2 resection status. By contrast, of the patients who underwent primary excision at our institute (*n* = 94), 83 % had R0 resection status, 13.8 % had R1 resection status, and 3.2 % had R2 resection status. The resection status of referred patients at presentation was significantly inferior to that of patients treated exclusively at our CCC (Chi^2^ test, *p* < 0.0001). After re-excision, 93.8 % of the referred patients had R0 resection status and 6.3 % had R1 resection status

Histopathological findings for the 71 patients who were referred to us after an unplanned excision and who had complete presentation status showed that 16.7 % had complete tumor resection (R0 resection status), 69.8 % had microscopic incomplete resection (R1 resection status), and 13.5 % had macroscopic incomplete resection (R2 resection status). By contrast, the data for patients treated exclusively at our institute showed that, after primary tumor excision, 83 % had complete tumor resection, 13.8 % had microscopic incomplete resection, and 3.2 % had macroscopic incomplete resection (*n* = 94) (Fig. 1).

Comparing both groups reveals several notable differences (Table 1). Tumors treated exclusively at our institution were more often high-grade tumors (Chi^2^ test, *p* = 0.019), larger than 5 cm (Chi^2^ test, *p* < 0.001), and had a higher National Comprehensive Cancer Network (NCCN) stage (Chi^2^ test, *p* < 0.001). However, there was no difference in the incidence of histological diagnosis (Chi^2^ test, *p* = 0.127). Although larger and higher-grade tumors were treated exclusively at our institute, the number of successful primary excisions was significantly higher (Chi^2^ test, *p* < 0.0001). For those who were initially treated elsewhere and had their resection status redefined after re-excision, the results were comparable to the group who were treated exclusively at our institute: we found complete resection in 93.8 % of cases, microscopic incomplete resection in 6.3 % of cases, and no cases of macroscopic incomplete resection (Fig. 1). Resection status was improved in 69 % of cases.

**Table 1:** Demographic and clinical data of referred patients and patients treated exclusively at our CCC

	CCC patients (n=94)	Referred patients with unplanned excision (n=71)	p-value	Statistical test
Demographics				Students *t* test
Male	47.9%	53.1%	0.469	
Female	52.1%	46.9%		
Under 50 years of age	27.7%	33.3%	0.396	
50 years of age and older	72.3%	66.7%		
Median age	59.49	55.94	0.156	
Clinical data				
FNCLCC grade				Chi^2^ test
Low	23.0%	23.5%	***0.019***	
Intermidiate	21.8%	40.0%		
High	55.2%	36.5%		
Tumour size				Chi^2^ test
Size ≤ 5 cm (T1)	30.2%	51.8%	***<0.001***	
Size > 5 cm (T2)	62.4%	48.2%		
Tumour location with regard to fascia				Chi^2^ test
Superficial (Ta)	32.2%	34.1%	0.788	
Deep (Tb)	67.8%	65.9%		
NCCN stage				Chi^2^ test
IA	4.6%	12.9%	***<0.001***	
IB	24.1%	15.3%		
IIA	12.6%	43.5%		
IIB	14.9%	9.4%		
III	36.8%	14.1%		
IV	6.9%	4.7%		
Histopathological diagnosis				Chi^2^ test
Liposarcoma	27.4%	37.3%	0.127	
Leiomyosarcoma	4.8%	4.8%		
Rhabdomyosarcoma	2.4%	3.6%		
Synovial sarcoma	4.8%	4.8%		
Malignant fibrous histiocytoma	33.3%	21.7%		
Fibrosarcoma	8.3%	13.3%		
Other	19.0%	14.5%		

### Residual tumor

Histopathological evidence of residual tumor after re-excision was found in 38 (53.5 %) of the referred patients who had undergone a “whoops” procedure (*n* = 71). As mentioned above, cases were coded according to their primary resection status—resection status after re-excision—and the existence of residual tumor at re-excision.

Our investigations of the reliability of primary resection status showed that half of the cases referred to us with supposed complete tumor resection showed evidence of residual tumor after revisional surgery (R0-R0-1, *n* = 6). On the other hand, no evidence of residual tumor was found in 27 cases that were initially said to have residual tumor (R1-R0-0 *n* = 25; R2-R0-0 *n* = 2). Primary histopathological resection status was thus misleading in nearly half of the referred cases (*n* = 33, 46.5 %). The six cases that presented with complete tumor resection but showed residual tumor in revisional surgery (R0-R0-1) very clearly demonstrate the potentially disastrous consequences of a misleading histopathological assessment. However, in the other 27 cases, revisional surgery allowed an ostensible revision of resection status and prevented adjuvant overtreatment.

On the other hand, revisional surgery that excised the residual tumor led to a real improvement in resection status in half of the cases (*n* = 35, 49.3 %; R0-R0-1 *n* = 6, R1-R0-1 *n* = 22, R2-R0-1 *n* = 6, and R2-R1-1 *n* = 1). The surgery truly improved local tumor control in these cases. The results definitely support a liberal indication for revisional surgery in cases of unplanned sarcoma excision.

**Fig. 2:**
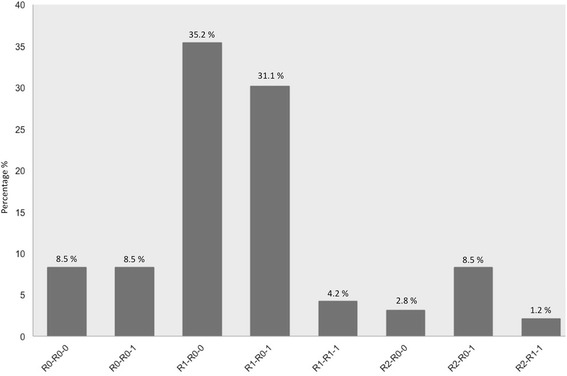
Distribution of referred patients after coding. Referred patients were categorized using the following code: 1. Primary resection status: R0, R1, or R2. 2. Resection status after re-excision: R0, R1, or R2. 3. Presence or absence of tumor at re-excision: 0 = no tumor found; 1 = tumor found. Overall, residual tumor after re-excision was found in 53.13 % of cases. Of all the patients referred with histopathological information indicating complete tumor resection, half had residual tumor in the re-excision specimen. In 33 cases (46.5 %), the primary histopathological information on resection status was misleading and either overestimated or underestimated the resection margins (R1-R0-0; R2-R0-0; R0-R0-1). In 27 cases (38 %), revisional surgery ostensibly improved resection status (R1-R0-0; R2-R0-0). In 35 cases (49.3 %), there was a real improvement (R0-R0-1; R1-R0-1; R2-R0-1; R2-R1-1). In nine cases (12.7 %), our surgery did not improve the resection status (R0-R0-0; R1-R1-1)

In some cases, revisional surgery did not improve resection status (R0-R0-0 *n* = 6, R1-R1-1 *n* = 3, 12.7 %) (Fig. 2). Our revisional surgery involved performing a simple re-excision with the aim of achieving a safety margin (*n* = 19), a wide excision (*n* = 41), a compartment resection (*n* = 7), a major amputation (*n* = 2), and a marginal excision (*n* = 2). We found that primary tumors located in an upper extremity were an independent risk factor for residual tumor (*p* = 0.001) (Table 2).

**Table 2:** Demographic and clinical data of referred patients with and without residual tumour at re-excision

	Referred patients without residual tumour (n=33)	Referred patients with residual tumour (n=38)	p-value	Statistical test
Demographics				Students *t* test
Male	57.8%	49.0%	0.391	
Female	42.2%	51.0%		
Under 50 years of age	75.6%	58.8%	0.083	
50 years of age and older	24.4%	41.2%		
Median age	57.24	54.78	0.523	
Clinical data				
FNCLCC grade				Chi^2^ test
Low	29.3%	18.2%	0.483	
Intermediate	36.6%	43.2%		
High	34.1%	38.6%		
Tumour size				Chi^2^ test
Size ≤ 5 cm (T1)	48.8%	54.5%	0.595	
Size > 5 cm (T2)	51.2%	45.5%		
Tumour location with reference to fascia				Chi^2^ test
Superficial (Ta)	39.0%	29.5%	0.357	
Deep (Tb)	61.0%	70.5%		
NCCN stage				Chi^2^ test
IA	17.1%	9.1%	0.146	
IB	17.1%	11.4%		
IIA	34.1%	47.7%		
IIB	9.8%	15.9%		
III	22.0%	9.1%		
IV	0.0%	6.8%		
Histopathological diagnosis				Chi^2^ test
Liposarcoma	37.8%	27.5%	0.861	
Leiomyosarcoma	6.7%	7.8%		
Rhabdomyosarcoma	2.2%	3.9%		
Synovial sarcoma	6.7%	7.8%		
Malignant fibrous histiocytoma	22.2%	15.7%		
Fibrosarcoma	11.1%	11.8%		
Other	13.30%	25.50%		
Location				Chi^2^ test
Upper extremity	17.8%	41.2%	***0.001***	
Lower extremity	62.2%	33.3%		
Head and neck	8.9%	0.0%		
Trunk	11.1%	25.5%		

### Local and systemic tumor control

**Fig. 3:**
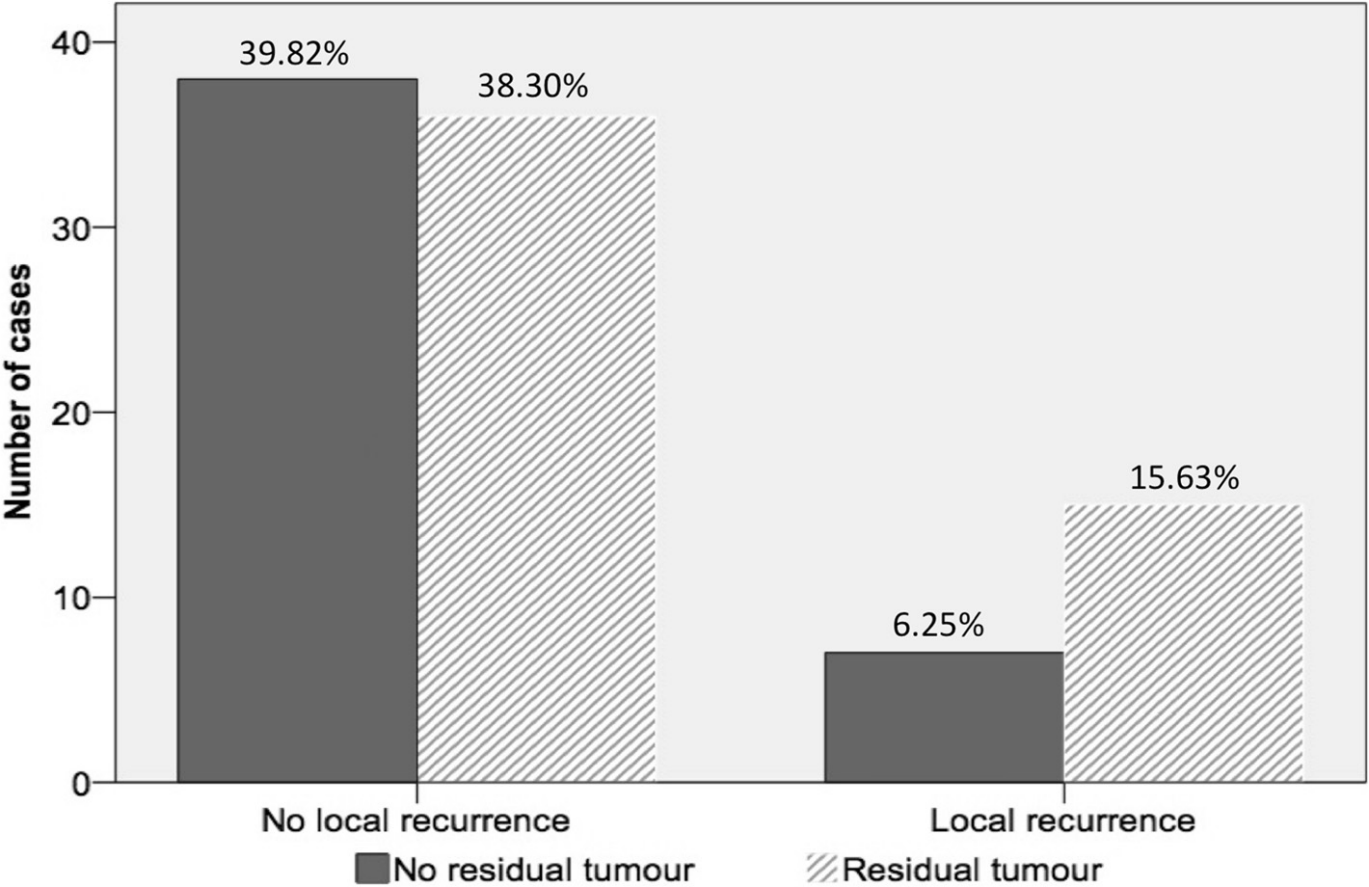
Local recurrence rate. We compared the local recurrence rate of the referred patients based on the presence or absence of residual tumor at re-excision (Chi^2^ test). The local recurrence rate after re-excision of residual tumor was 15.63 % (11 cases), compared to 6.25 % (4 cases) when no residual tumor was found. Although the difference was not significant, there was a strong tendency towards significance (*p* = 0.057)

**Fig. 4:**
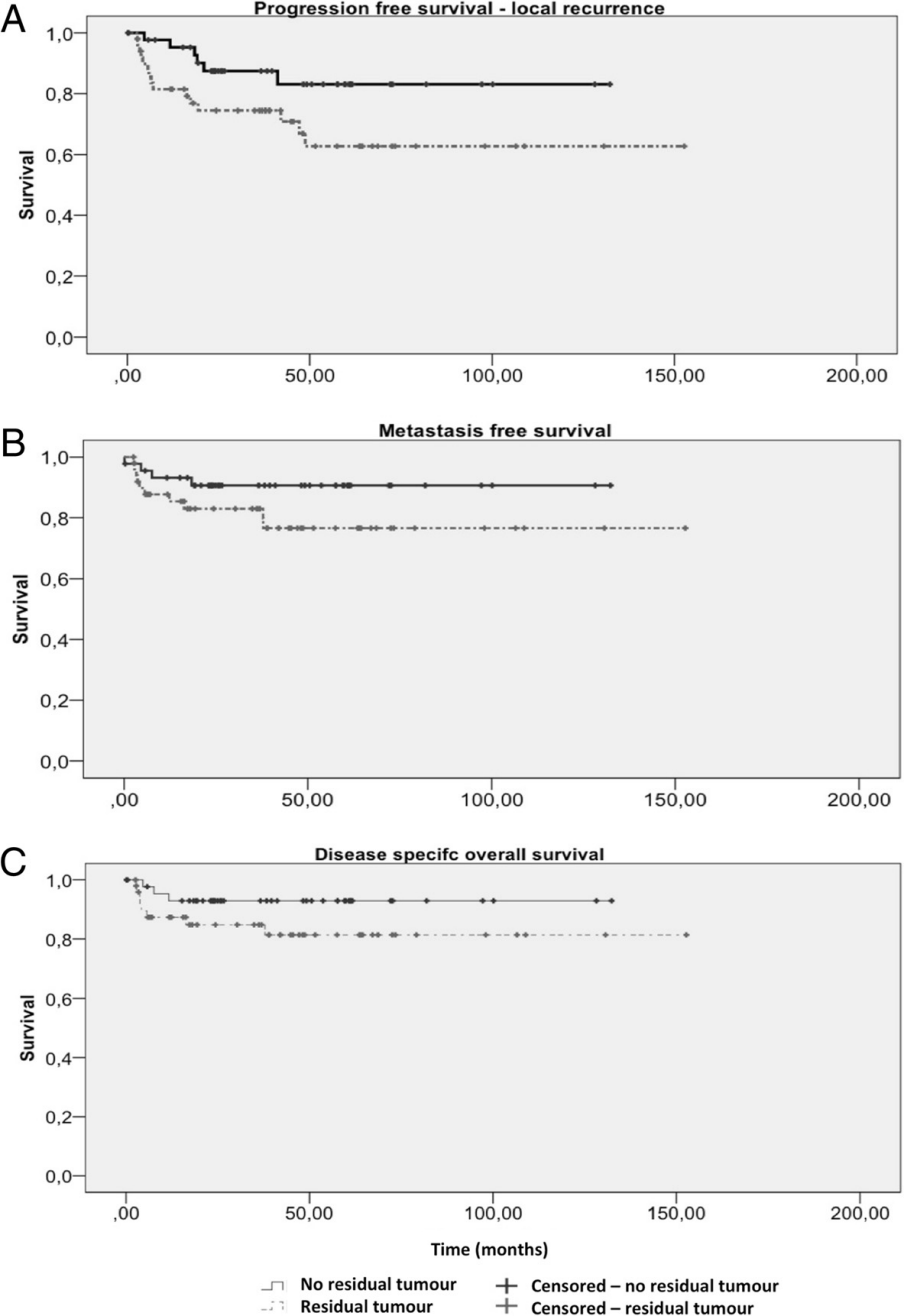
Progression-free survival and disease-specific overall survival. We compared the referred patients based on the presence or absence of residual tumor at re-excision. We did so using a log-rank test for the following items: **a** Recurrence-free survival: Time was significantly shorter in cases where residual tumor existed (*p* < 0.05). [Five-year recurrence-free survival (±standard error): 83 ± 6 % without residual tumor at re-excision; 63 ± 8 % with residual tumor]. **b** Metastasis-free survival: No significant difference (*p* = 0.127). [Five-year metastasis-free survival (±standard error): 90 ± 4.5 % without residual tumor at re-excision; 76.6 ± 6.7 % with residual tumor]. **c** Disease-specific overall survival: Groups were comparable (*p* = 0.137). [Five-year disease-specific survival (±standard error): 92.9 ± 3.9 % without residual tumor at re-excision; 81.4 ± 6.1 % with residual tumor]

Residual tumor might be a risk factor for local recurrence as it approaches significance (15.63 % when residual tumor found vs. 6.25 % when no residual tumor found; *p* = 0.057; Chi^*2*^ test; Fig. 3). At the same time, progression-free survival was significantly reduced in cases of residual tumor (5 years of progression-free survival: 83 ± 6 % vs. 63 ± 8 %; log-rank test; *p* < 0.05). The rate of metastasis was comparable (10.4 vs. 4.2 %; log-rank test; *p* = 0.138) without significant difference in metastasis-free survival (90 ± 4.5 % vs. 76.6 ± 6.7 % at 5 years; log-rank test; *p* = 0.127) or in disease-specific survival (92.9 ± 3.9 % vs. 81.4 ± 6.1 % at 5 years; log-rank test; *p* = 0.137; Fig. 4).

## Discussion

Our CCC serves as a referral center for soft tissue sarcomas. Of the patients treated here, more than one in two were referred to us after undergoing primary surgery in a non-CCC setting. Nearly three-quarters underwent a “whoops” procedure. This experience is consistent with that of other authors [1, 2, 31–33]. The situation is caused by the rarity of this heterogeneous group of malignant mesenchymal tumors and by the fact that they can occur anywhere in the body [12, 13, 34, 35]. Furthermore, their clinical behavior is characterized by slow growth, they cause few physical complaints at first and they can appear harmless in cross-sectional imaging—all of which can be misleading [36]. Certain anatomical regions can pose a particular challenge. This is the case with uterine sarcomas, for instance. They are very rare, the symptoms are misleading, and the lack of pathognomonic clinical or diagnostic signs means that delayed therapy is the rule [37]. Soft tissue sarcomas are underestimated at different stages of treatment. An insufficient diagnostic and staging workup, including biopsy type and execution, could have fatal consequences. A lack of experience in specific treatment or plastic surgical reconstruction techniques can lead to insufficient primary tumor excision and disastrous consequences [3, 4, 14, 18–21, 38].

Our data clearly show that, when an unplanned sarcoma excision is performed in a non-CCC setting, the initial histopathological assessments are misleading and should not be used as a basis for consultations and treatment planning. In half of the cases, initial and final resection statuses were conflicting and in some instances resulted in mutilating surgery that may not have been necessary. Histopathological assessments of sarcomas are complex and challenging. Reliability depends on experience and an institute’s caseload [27, 28, 39]. Higher caseloads at CCCs and regular reviews of histopathological assessments by external experts enhance reliability. However, a lack of sufficient number of cases for statistical evaluation (2 out of 94) meant that we could not compare the reliability of histopathological results after revisional surgery in patients treated exclusively at our center and those treated out of a CCC. Furthermore, the conclusions drawn from our results are limited by the fact that the patients who were referred to our center for further treatment were pre-selected. It is likely that many patients who supposedly had complete tumor resection were not referred for further treatment or re-assessment.

A primary tumor located in an upper extremity is an individual risk factor for residual tumor. This is not surprising, as the close anatomical relationship between functionally relevant structures reduces the likelihood of radicality during an unplanned excision. A lack of expertise in reconstructive surgery and defect coverage is another factor that often causes uncertainty and restraint in unplanned excisions.

This uncertainty and restraint is also indirectly reflected in the radicality achieved in our center, where revisional surgery improved the resection status in 69 % of the referred cases. Radicality can be increased in an interdisciplinary setting where surgeons have experience of reconstruction. Although the patients who were treated exclusively at our center had significantly more high-grade and larger tumors at a more advanced NCCN stage, the radicalism achieved was initially superior and comparable after revisional surgery.

Residual tumor seems to be a prognostic factor for significantly earlier local tumor recurrence and shows a tendency towards an increased risk of local recurrence. Our results are in line with Gustafson et al., who showed that the number of operations required and the local recurrence rate was higher in referred patients and in patients who did not receive any treatment in a CCC [32]. Furthermore, Kang et al. identified the level of the referring hospital as a risk factor for oncological outcomes [38].

However, our results conflict with the results of other studies that show sufficient local tumor control but an increased rate of metastasis after adequate re-excision [16, 25]. Lewis et al. published surprising data that indicated that additional wide resection after an unplanned excision was an independent, favorable variable that improved overall survival compared to primary tumor excision at their cancer center [24]. Other studies showed an increased local recurrence rate and metastasis rate even after re-excision [6, 18, 26]. Morii et al. found that additional wide resection is the only therapy option that improves oncological outcomes [23]. However, delayed re-excision seems to have no influence on oncological outcomes [40].

In this study, we performed a retrospective chart review, a study design in which research questions are answered by analyzing pre-coded, patient-centered data. The design has several drawbacks compared to prospective studies. Firstly, there is often a failure to formulate a well-defined, clearly articulated research question before the chart review begins. Relevant answerable questions are often limited by the quality and amount of pre-coded data. Secondly, operationalization of the data and standardization of data abstraction is limited, which means accuracy and consistency are also limited. Thirdly, the person doing the abstraction is generally not blinded to the hypothesis and the cases, which leads to a reviewer bias. Notwithstanding these limitations, retrospective case studies are a relevant study design for producing information on rare diseases. In the case of such a heterogeneous and rare disease as soft tissue sarcomas, the only way to plan and successfully perform a prospective study is via a multicenter design.

## Conclusions

An unplanned excision of a soft tissue sarcoma should always be followed by re-excision. Resection margins and histopathological assessments performed by low-volume hospitals are unreliable and should not be taken as the basis for treatment planning. Excisions performed at high-volume centers are more radical than those done at referring centers. Residual tumor at re-excision is a risk factor for earlier and more likely local recurrence.

## Abbreviations

AWD: alive with disease
CCC: comprehensive cancer center
DOC: dead of other causes
DOD: dead of disease
FNCLCC: Federation Nationale des Centres de Lutte Contre le Cancer
MRI: magnetic resonance imaging
NCCN: National Comprehensive Cancer Network
NED: no evidence of disease
STS: soft tissue sarcoma

## References

[1] Bannasch H, Haivas I, Momeni A, Stark GB (2009). Oncosurgical and reconstructive concepts in the treatment of soft tissue sarcomas: a retrospective analysis. Arch Orthop Trauma Surg.

[2] Bhangu AA, Beard JA, Grimer RJ (2004). Should soft tissue sarcomas be treated at a specialist centre?. Sarcoma.

[3] Davis AM, Kandel RA, Wunder JS, Unger R, Meer J, O’Sullivan B (1997). The impact of residual disease on local recurrence in patients treated by initial unplanned resection for soft tissue sarcoma of the extremity. J Surg Oncol.

[4] Noria S, Davis A, Kandel R, Levesque J, O’Sullivan B, Wunder J (1996). Residual disease following unplanned excision of soft-tissue sarcoma of an extremity. J Bone Joint Surg Am.

[5] Rydholm A (1998). Improving the management of soft tissue sarcoma. Diagnosis and treatment should be given in specialist centres. BMJ.

[6] Siebenrock KA, Hertel R, Ganz R (2000). Unexpected resection of soft-tissue sarcoma. More mutilating surgery, higher local recurrence rates, and obscure prognosis as consequences of improper surgery. Arch Orthop Trauma Surg.

[7] Lehnhardt M, Daigeler A, Homann HH, Hauser J, Langer S, Steinstrasser L (2009). Importance of specialized centers in diagnosis and treatment of extremity-soft tissue sarcomas. Review of 603 cases. Der Chirurg Zeitschrift fur alle Gebiete der operativen Medizen.

[8] Gutierrez JC, Perez EA, Moffat FL, Livingstone AS, Franceschi D, Koniaris LG (2007). Should soft tissue sarcomas be treated at high-volume centers? An analysis of 4205 patients. Ann Surg.

[9] Bannasch H, Eisenhardt SU, Grosu AL, Heinz J, Momeni A, Stark GB (2011). The diagnosis and treatment of soft tissue sarcomas of the limbs. Dtsch. Arztebl. Int.

[10] Steinau HU, Steinstrasser L, Hauser J, Tilkorn D, Stricker I, Daigeler A (2012). Soft tissue sarcoma: resection and plastic reconstruction. Der Chirurg Zeitschrift fur alle Gebiete der operativen Medizen.

[11] Nystrom LM, Reimer NB, Reith JD, Dang L, Zlotecki RA, Scarborough MT (2013). Multidisciplinary management of soft tissue sarcoma. ScientificWorldJournal.

[12] Jane MJ, Hughes PJ (1998). Disease incidence and results of extremity lesion treatment: Mersey region soft tissue sarcomas (1975–1985). Sarcoma.

[13] Jemal A, Tiwari RC, Murray T, Ghafoor A, Samuels A, Ward E (2004). Cancer statistics, 2004. CA Cancer J Clin.

[14] Giuliano AE, Eilber FR (1985). The rationale for planned reoperation after unplanned total excision of soft-tissue sarcomas. J. Clin. Oncol. Off. J. Am. Soc. Clin. Oncol.

[15] Steinau HU, Steinstrasser L, Langer S, Stricker I, Goertz O (2011). Surgical margins in soft tissue sarcoma of the extremities. Pathologe.

[16] Karakousis CP, Driscoll DL (1999). Treatment and local control of primary extremity soft tissue sarcomas. J Surg Oncol.

[17] Clark MA, Fisher C, Judson I, Thomas JM (2005). Soft-tissue sarcomas in adults. N Engl J Med.

[18] Umer HM, Umer M, Qadir I, Abbasi N, Masood N (2013). Impact of unplanned excision on prognosis of patients with extremity soft tissue sarcoma. Sarcoma.

[19] Chandrasekar CR, Wafa H, Grimer RJ, Carter SR, Tillman RM, Abudu A (2008). The effect of an unplanned excision of a soft-tissue sarcoma on prognosis. J. Bone Joint Surg.

[20] Fiore M, Casali PG, Miceli R, Mariani L, Bertulli R, Lozza L (2006). Prognostic effect of re-excision in adult soft tissue sarcoma of the extremity. Ann Surg Oncol.

[21] Venkatesan M, Richards CJ, McCulloch TA, Perks AG, Raurell A, Ashford RU (2012). Inadvertent surgical resection of soft tissue sarcomas. Eur. J. Surg. Oncol.

[22] Alamanda VK, Delisca GO, Mathis SL, Archer KR, Ehrenfeld JM, Miller MW (2013). The financial burden of reexcising incompletely excised soft tissue sarcomas: a cost analysis. Ann Surg Oncol.

[23] Morii T, Yabe H, Morioka H, Anazawa U, Suzuki Y, Toyama Y (2008). Clinical significance of additional wide resection for unplanned resection of high grade soft tissue sarcoma. Open Orthop J.

[24] Lewis JJ, Leung D, Espat J, Woodruff JM, Brennan MF (2000). Effect of reresection in extremity soft tissue sarcoma. Ann Surg.

[25] Rehders A, Stoecklein NH, Poremba C, Alexander A, Knoefel WT, Peiper M (2009). Reexcision of soft tissue sarcoma: sufficient local control but increased rate of metastasis. World J Surg.

[26] Hays DM, Lawrence W, Wharam M, Newton W, Ruymann FB, Beltangady M (1989). Primary reexcision for patients with ‘microscopic residual’ tumor following initial excision of sarcomas of trunk and extremity sites. J Pediatr Surg.

[27] Harris M, Hartley AL (1997). Value of peer review of pathology in soft tissue sarcomas. Cancer Treat Res.

[28] Harris M, Hartley AL, Blair V, Birch JM, Banerjee SS, Freemont AJ (1991). Sarcomas in north west England: I. Histopathological peer review. Br J Cancer.

[29] Hasegawa T, Yamamoto S, Nojima T, Hirose T, Nikaido T, Yamashiro K (2002). Validity and reproducibility of histologic diagnosis and grading for adult soft-tissue sarcomas. Hum Pathol.

[30] Kaste SC, Hill A, Conley L, Shidler TJ, Rao BN, Neel MM (2002). Magnetic resonance imaging after incomplete resection of soft tissue sarcoma. Clin Orthop Relat Res.

[31] Alamanda VK, Crosby SN, Archer KR, Song Y, Schwartz HS, Holt GE (2012). Primary excision compared with re-excision of extremity soft tissue sarcomas--is anything new?. J Surg Oncol.

[32] Gustafson P, Dreinhofer KE, Rydholm A (1994). Soft tissue sarcoma should be treated at a tumor center. A comparison of quality of surgery in 375 patients. Acta Orthop Scand.

[33] Steinau HU, Homann HH, Drucke D, Torres A, Soimaru D, Vogt P (2001). Resection method and functional restoration in soft tissue sarcomas of the extremities. Der Chirurg Zeitschrift fur alle Gebiete der operativen Medizen.

[34] Lahat G, Tuvin D, Wei C, Anaya DA, Bekele BN, Lazar AJ (2008). New perspectives for staging and prognosis in soft tissue sarcoma. Ann Surg Oncol.

[35] Lahat G, Lazar A, Lev D (2008). Sarcoma epidemiology and etiology: potential environmental and genetic factors. Surg. Clin. North Am.

[36] Berger F, Winkler EC, Ruderer C, Reiser MF (2009). Imaging of soft tissue sarcomas: standard approaches and new strategies. Der Chirurg Zeitschrift fur alle Gebiete der operativen Medizen.

[37] Papadia A, Salom EM, Fulcheri E, Ragni N (2007). Uterine sarcoma occurring in a premenopausal patient after uterine artery embolization: a case report and review of the literature. Gynecol Oncol.

[38] Kang S, Han I, Lee SA, Cho HS, Kim HS (2013). Unplanned excision of soft tissue sarcoma: the impact of the referring hospital. Surg Oncol.

[39] Ray-Coquard I, Montesco MC, Coindre JM, Dei Tos AP, Lurkin A, Ranchere-Vince D (2012). Sarcoma: concordance between initial diagnosis and centralized expert review in a population-based study within three European regions. Ann. Oncol ESMO.

[40] Han I, Kang HG, Kang SC, Choi JR, Kim HS (2011). Does delayed reexcision affect outcome after unplanned excision for soft tissue sarcoma?. Clin Orthop Relat Res.

